# The Need for a Paradigm Shift in Approaching Ageing-Related Design Research and Practice

**DOI:** 10.3389/fpsyg.2021.750178

**Published:** 2021-11-02

**Authors:** Elena Comincioli, Alice Chirico, Andrea Gaggioli, Masood Masoodian

**Affiliations:** ^1^School of Arts, Design and Architecture, Aalto University, Espoo, Finland; ^2^Catholic University of the Sacred Heart, Milano, Italy; ^3^Department of Psychology, Catholic University of the Sacred Heart, Milano, Italy

**Keywords:** healthy ageing, healthy ageing framework, positive psychology, implicit ageism, design for ageing, ageing society, ageing population, ageism and age-based discrimination

## Abstract

Despite a rapidly ageing world population, ageism – particularly in its implicit form – is widespread in society. In this article, we propose that a paradigm shift is needed in how we approach ageing-related design research and practice in areas such as assistive technologies. We also put forward the idea of using the Healthy Ageing (HA) framework of the WHO as the basis for new lines actions that can be taken by design researchers and practitioners to address implicit ageism in society through their work.

## Introduction

The world population is rapidly ageing. It is predicted that, for instance, people aged 65years and over will represent 45% of the population of Europe by 2070 (Directorate-General for Economic and Financial Affairs, 2018). Although, this forecast is likely to become a reality, ageism – defined as “an alteration in feeling, belief, or behaviors in response to an individual’s or group’s perceived chronological age” (Levy and Banaji, 2002) – is sadly a common experience for most ageing people (Ayalon and Tesch-Römer, 2018). Such experiences can in turn have detrimental effects on older adults’ health and well-being (Levy and Banaji, 2002) – e.g., leading to higher blood pressure, reduced self-esteem and motivation, and lower life expectancy (Levy et al., 2000, 2002). Stereotypes and biases about old age are, however, so pervasive that even older adults themselves often can have such views (Coughlin, 2017; Voss et al., 2017). Therefore, many negative norms of an ageist society can generally be difficult to identify and challenge (Carstensen, 2011; Applewhite, 2016).

Fortunately, targeting ageism and improving the health and well-being of older adults (Burns and Masoodian, 2018) have in recent years become important issues to address for researchers and practitioners from a range of disciplines. An increasingly used framework for addressing ageism is that of Healthy Ageing (HA). While this terminology has started to appear in related literature since at least the year 2000, its meaning has changed over the years, along with the ongoing debate on the broader definition of health itself (Sholl and Rattan, 2020). Such discussions surrounding health can be divided into two competing approaches: (1) health considered simply as the absence of diseases, or (2) health as “a state of complete physical, mental, and social well-being and not merely the absence of disease or infirmity” (WHO, 2006). The second approach is proposed by the WHO, which also introduced its related HA framework in 2015.

With recent developments in fields such as Artificial Intelligence, Intelligent Environments, and Internet of Things, assistive technologies are seen as potential platforms for improving health and well-being of ageing people (Jacobson, 2014; Lee and Riek, 2018). While such advanced technologies are opening up many new possibilities in this direction, more often than not most their designs target solely the health “needs” of older adults (Burns and Masoodian, 2018) or mainly aim to solve various “problems” associated with ageing (Comincioli et al., 2021). When such approaches are taken, all the other elements of HA – for instance, the intrinsic capabilities of older adults and their well-being – tend to be neglected, and ageist ideas and assumptions tend to guide the design, development, deployment, and evaluation of assistive technologies. To combat these tendencies, here, we adopt the WHO HA framework as the basis for proposing lines of action that support a paradigm shift in approaching ageing-related research and practice in field of assistive technologies in particular, as well as other areas of product and service design in general.

## Healthy Ageing Framework

The HA framework calls for a radical change in how society thinks, talks, and acts toward ageing (WHO, 2020). According to WHO, HA is “*the process of developing and maintaining the functional ability that enables well-being in older age*” (World Health Organization, 2021). In this definition, *functional abilities* are related to both internal and external factors – from each individual’s *intrinsic capacity* to *environmental conditions*, and the interactions between these elements. While *intrinsic capacity* refers to “*all the mental and physical capacities that a person can draw on*” (World Health Organization, 2021), environmental variables account for all levels, from macro to mezzo and micro, including “*the home, community, and broader society, and all the factors within them such as the built environment, people and their relationships, attitudes and values, health and social policies, the systems that support them and the services that they implement*” (World Health Organization, 2021).

The aim of the HA framework is to tackle four main challenges that societies with ageing populations face, particularly in promoting better design and improved access to quality services for older adults. These challenges are:

- “Diversity in older age” (World Health Organization, 2021) posits that age is only an indicator, which on its own does not say much about a person’s mental or physical abilities. It rejects the idea that it is possible to form a full picture of a typical older adult based on age (Carstensen, 2011; Coughlin, 2017; World Health Organization, 2021), and encourages researchers and practitioners to examine the life experiences of individuals in order to understand their personal needs and desires better – e.g., instead of using statistics to define a group of older adults, define more specific subgroups, as done by sociologists (Komp and Aartsen, 2013).
- “Health inequities” (World Health Organization, 2021) requires looking at the relationship between health and age from a broader perspective, by acknowledging the role of environmental factors – e.g., those relating to the family, gender, and ethnicity of individuals and the social context in which they have lived through all their life stages.
- “A rapidly changing world” (World Health Organization, 2021) means that it is necessary to consider the macro trends shaping the contemporary world – for instance, globalization, urbanization, social migration, and changing gender norms (World Health Organization, 2021). Particular attention should be given to how “technological, scientific, medical (including new treatments) assistive technologies and digital innovation (…) can foster Healthy Ageing” (WHO, 2020).
- “Outdated and ageist stereotypes” (World Health Organization, 2021) relate to how older adults are depicted as frail, dependent, or generally an economic burden on society. WHO recognizes that the pervasiveness of these ideas is such that they can lead to discrimination, and hinder the development of social policies and opportunities targeted at older people (World Health Organization, 2021). Any discrimination based on age also poses a barrier to research and practice, because ageism “influences the way problems are framed, and the question asked, and the solution offered” (WHO, 2020). This challenge is, therefore, the most relevant to our discussion here.

## Forms of Ageism

Ageism can take many forms, and ageist biases can be found at different levels in society. At a *macro level*, ageism can appear in the form of an ageist perspective – e.g., in the beauty industry, where the term anti-aging is used to portray ageing as something to be avoided (Levy and Banaji, 2002). At a *micro level*, on the other hand, ageism can appear in our language – e.g., to express discrimination and contempt toward old age and older adults (Gendron et al., 2016).

What makes ageism rather difficult to address is that “[it] can operate without conscious awareness, control, or intention to harm” (Levy and Banaji, 2002). As such, the concept of “implicit ageism” is often used to highlight the deceptive nature of this form of discrimination. Implicit ageism can be in the form of stereotypes, attitudes, or biases. *Implicit ageist stereotypes* are “thoughts about the attributes and behaviors of the elderly that exist and operate without conscious awareness, intention, or control” (Levy and Banaji, 2002). *Implicit ageist attitudes* are “feeling toward the elderly that exist and operate without conscious awareness, intention, or control” (Levy and Banaji, 2002). *Implicit ageist biases* are prejudices and preconceived opinions about the elderly (for a general definition of biases, see Gendron et al., 2016). Stereotypes are, therefore, a form of bias, which show an individual’s thoughts, beliefs, and expectations regarding another individual without actually having any objective comprehension of the person in question (Fiske, 2014). Once a bias is formed in a person’s mind, it is hard to eradicate. Even if people are exposed to evidence that contradicts their biases, they are likely to treat such evidence as an exception, to the point that the evidence may even further confirm their false convictions (Levy and Banaji, 2002). While stereotypes are static entities, aiming to create order by disregarding any dynamism (Krekula, 2009), when they are used to make sense of the world, they perpetuate discrimination (Fiske, 2014).

For instance, a common implicit ageist stereotype is that older adults are not able to contribute to society, and therefore they are a valuable part of their communities, and are perceived as fragile and dependent – with the resulting prevalent social attitude toward older people being that of distancing (Levy and Banaji, 2002; Krekula, 2009). Such ageist ideas are often normed and tolerated by society (Gendron et al., 2016), or even encouraged and reinforced, for instance, through benevolent ageism (19, 20) or humor (ICAA, 2011) – e.g., in the use of “funny” ageist birthday cards (Ellis and Morrison, 2005) degrading older people. This is a significant difference between ageism and other forms of social discrimination, in that people expressing ageist remarks are rarely reprimanded, and as such, “ageism, unlike racism, does not provoke shame” (Levy and Banaji, 2002).

Another characteristic of ageism is that those who perpetrate it will sooner or later themselves be subjected to it – in other words, young people perpetrating ageist views discriminate toward their own future selves (Jönson, 2013). Such negative beliefs and attitudes toward old age are, however, formed from a young age (Vauclair et al., 2018) and seem to persist throughout a person’s entire life (Levy and Banaji, 2002). Moreover, this attitude characterizes the difference between discrimination based on age and those based for instance on race, gender, or religion. While the members of such other groups usually express a strong preference toward their peers (Levy and Banaji, 2002), ageism tends to be commonly self-inflicted – defined as intrinsic ageism (Levy and Banaji, 2002; Gendron et al., 2016). For instance, older adults with high self-esteem identify themselves with younger people rather than their own age peers (Greenwald et al., 2002).

## Targeting Ageism: a Call To Action

Many design researchers and practitioners developing products and services – such as assistive technologies – targeting issues relating to ageing often follow a clinical notion of ageing, which is generally based on a *deficit model* of ageing (Bangen et al., 2013). Unfortunately, not only this model makes some generalizations in line many existing stereotypes about ageing, most people following such a biased model do so without “conscious awareness, control, or intention to harm” (Levy and Banaji, 2002). Furthermore, the roots of prejudices that shape people’s ageist biases and stereotypes can usually be found at levels that are “unnoticed and uncontrollable” (Levy and Banaji, 2002).

Therefore, we believe that by initially targeting people in fields such as product and service design, whose research or practice is concerned with ageing, it may be more effective to raise societal awareness of the negative impacts of – often implicit – ageism and foster a paradigm shift in how age is addressed by society. As such, in this article, we call for all concerned design researchers and practitioners to consider their work as a strategic component that can actively combat ageism and challenge the status quo. To do so, we suggest three different lines of actions to target implicit ageism.

### Action 1: Changing the Language of Ageing

The HA framework of WHO calls for a transdisciplinary approach to tackle the four main challenges of an ageing society. A strategic step in this approach is the formation of a research network that brings together experts and researchers from different fields (WHO, 2020), to create a common ground and develop “an approach that crosses traditional disciplinary borders and critically extends a disease-focused methodology” (Kliegel et al., 2020).

A first step in establishing such a common ground is to redefine the shared language used in talking about ageing. According to Gendron et al. (2016), “language is the basis through which we communicate with each other. Through language, we share our thoughts, ideas, and emotions.” Zlatev and Blomberg (2015) have investigated the relationship between language and thought, and argue that the former can influence the latter. Following these ideas, we propose a change in lexicon commonly used when referring to ageing, by developing a more accurate and sensitive language about the reality of ageing (American Psychological Association, 2020). This would, in turn, encourage a shift in the way society thinks about this topic and approaches it – e.g., by critically challenging existing biases and stereotypes.

**Figure 1:**
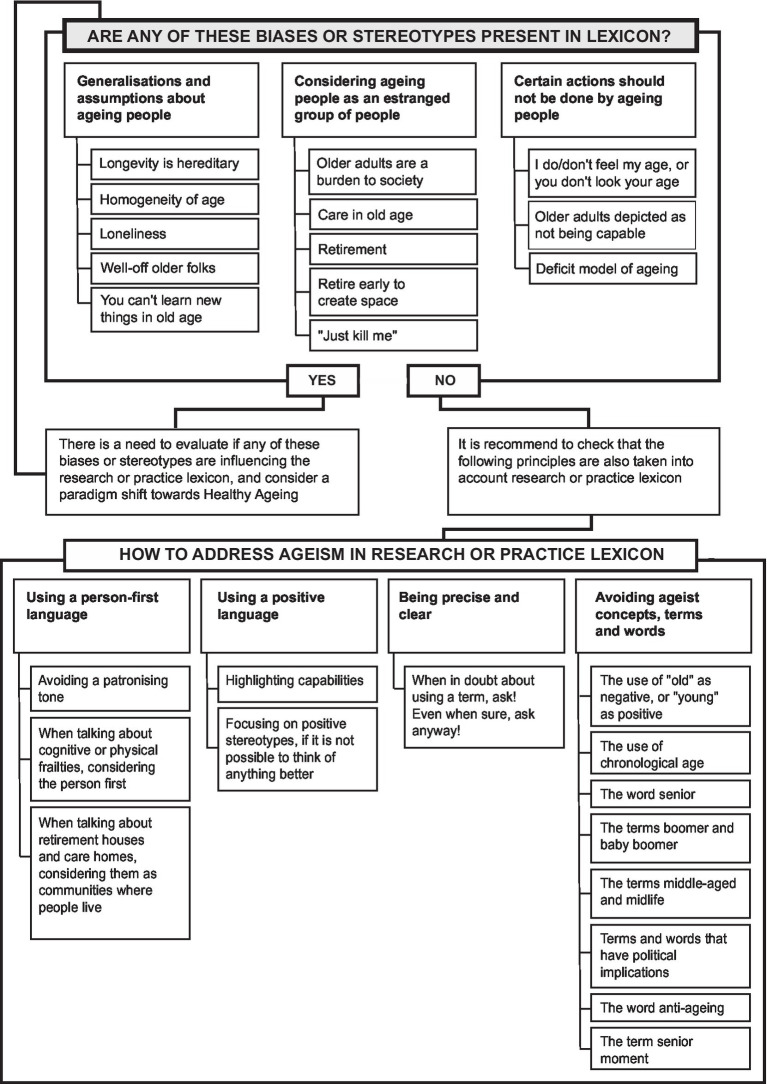
Summary of the guidelines to promote the use of an appropriate lexicon about ageing.

Since “long-standing cultural practice can exert a powerful influence over even the most conscientious writer” (American Psychological Association, 2020), here, we present an initial set of guidelines to promote the use of an appropriate lexicon about ageing. The aim of these guidelines, as presented in Figure 1, is to help researchers and practitioners to recognize any inappropriate lexicon they might be using in their work – often without knowing their underlying implicit ageist biases and stereotypes.

In addition, it is important to note that in terms of implicit ageism, particular attention should be given to the risk of using *false friends*. In linguistics, the term *false friends* is used to refer to words that sound similar in two languages but differ substantially in their meanings. In our context, *false friends* can be defined as using seemingly appropriate lexicon about ageing, which instead normalize ageist biases and stereotypes. Even if such *false friends* are used with the best of intentions, this can ultimately lead to opposite results by reinforcing implicit ageism.

### Action 2: Changing the Perspective on Ageing

To take an active step toward addressing ageism, design researchers and practitioners should aim to increase awareness of the importance of ageing people as an important segment of society, whose well-being, health, and quality of life must be improved. To this end, the approach promoted by Positive Psychology (PP) to consider health and well-being can be adopted. PP arose in the early 2000s from the enlightened thinking and actions of several scholars led by Seligman (2019), and builds upon the idea that people need to thrive and not just to survive. As such, according to PP, health and well-being cannot be reduced to the mere absence of diseases – in line with HA, as proposed by WHO – but rather, it should be understood as a way to pursue flourishing in life at all levels (Seligman, 2011).

In this salutogenic perspective rooted in seminal work of Antonovsky (1996), factors such as money, economics, and illnesses can contribute only to a small part of a person’s happiness and well-being (Park and Corn, 2017). Other positive elements of people’s existence – such as their emotions, relationships, accomplishments, and satisfaction with life – also contribute immensely to their health and well-being (Khaw and Kern, 2015) at any age (Diener and Seligman, 2004; Araujo et al., 2017). Therefore, in targeting ageism, society must value older adults’ strengths, abilities, and capabilities as factors affecting their well-being.

### Action 3: Changing the Experience of Ageing

Design researchers and practitioners can help to transform society by combating ageism through improving the experience of ageing in the lives of older adults. A practical approach toward this goal is by utilizing the Transformative Experience Design (TED) framework (Gaggioli et al., 2016). TED proposes a step-by-step process that can be adopted to generate interventions or case studies with transformative potential. The TED framework can help to create experiences that “can completely alter one’s relationship with the self-world: the individual builds up a new worldview, and this new perspective supports lasting change” (Gaggioli et al., 2016). TED focuses on four elements: (1) the *medium* used to deliver the experience, (2) the *content* of the experience itself, (3) the *form* relating to the style used to deliver the experience, and (4) the ultimate *purpose* or goal pursued by the designer (Gaggioli et al., 2016). The process starts by exposing the target audience – in our case, older adults – to new information – framed using the four elements, thus allowing them to start a process of assimilation of the new information, and challenging their initial worldview. In this way, the process produces a *critical fluctuation* that can result either in a *rejection of the novelty* or in an *accommodation of existing schemas* and generation of new knowledge (Gaggioli et al., 2016).

## Discussion and Conclusion

Over the past two decades, Human-Centered Design (HCD) methodologies have been promoted as the most effective approaches for designing better user experiences. At the core of HCD is the idea of focusing on the needs of potential users of the intended design. As such, when HCD methodologies have guided the design of – especially digital – products, tools, and services for ageing people, the aim has often been to address the needs of older users by attempting to solve their ageing-related “problems” (Vines et al., 2015). This approach has, in turn, resulted in certain implicit stereotypes and biases becoming dominant in design research and practice targeted at ageing people, particularly in technical fields such Assistive Technology. Since our future world and society is shaped by such technological innovations and designs, the role that their design researchers and practitioners play in addressing existing biases and stereotypes toward ageing is of outmost importance.

Therefore, we believe that by adopting the HA framework as the basis for a paradigm shift in how ageing is addressed in design research and practice – as well as in other fields dealing with ageing – substantial advancements can be made in society toward combating implicit ageism. To that end, this perspective is meant as a call to action for such scholars and experts from related disciplines by helping them to shift their perspective from a *deficit model of ageing* toward a more salutogenic approach, so that they can take an active role in fighting ageism in larger society. As a starting initiative, we have thus presented three separate lines of action that can be followed by such researchers and practitioners in achieving this goal. By sharing these ideas, we ultimately aim to promote further debate, and provide alternative – or complimentary – future lines of actions.

It must also be noted here that this perspective cannot – and is not intended to – offer guidelines that all researchers and practitioners can blindly adopt and follow. Instead, the intention is that each perspective reader should determine to what extend changes can be made in their respective field. However, we also believe that the guidelines provided in this article can help those working particularly in transdisciplinary teams, in which people who are from different fields – such as design, technology, psychology, and affective sciences – can share a common language devoid of ageist stereotypes in addressing challenges and utilizing opportunities provided by an ageing society. These guidelines may, on the other hand, be rather difficult to adopt and fully apply in disciplines investigating, for instance, clinical or biological aspects of the aging process, which might require their own specific language or approaches to research and practice.

Finally, it should be pointed out that, as with all stereotypes and biases, things are not always black and white, and there are many existing and emerging nuances in terms of ageism as well. In this respect, culture also plays a crucial role in how stereotypes and biases are shaped, perpetuated, and normed in society, thus influencing widespread perceptions of ageing (Vauclair et al., 2017). As a result, in some cultures benevolent forms of ageism are more common than the malevolent forms (Cary et al., 2017). It is also true that benevolent ageism can be helpful to researchers and practitioners who might find it difficult to shift their perspective from a deficit model of ageing to one based on the HA framework, and as such, they may find benevolent ageism as an initial starting point for a change of paradigm in approaching ageing discourse (Comincioli et al., 2021). Nevertheless, it is important to note that both forms of ageism – with different effects – are ultimately detrimental to the health and well-being of ageing people (Levy and Banaji, 2002). Therefore, the intention of our work is to provide guidelines to design researchers and practitioners on how to address ageism in all its forms when adopting the HA framework.

## Data Availability Statement

The original contributions presented in the study are included in the article/supplementary material, further inquiries can be directed to the corresponding author.

## Author Contributions

EC and AC contributed to the conception and design of the perspective. EC wrote the first draft of the manuscript. AC, AG, and MM wrote sections of the manuscript. All authors contributed to the article and approved the submitted version.

## Conflict of Interest

The authors declare that the research was conducted in the absence of any commercial or financial relationships that could be construed as a potential conflict of interest.

## Publisher’s Note

All claims expressed in this article are solely those of the authors and do not necessarily represent those of their affiliated organizations, or those of the publisher, the editors and the reviewers. Any product that may be evaluated in this article, or claim that may be made by its manufacturer, is not guaranteed or endorsed by the publisher.

## References

[ref1] American Psychological Association (2020). Publication Manual of the American Psychological Association. 7th Edn. Washington, DC: American Psychological Association (APA).

[ref2] Antonovsky A. (1996). The salutogenic model as a theory to guide health promotion. Health Promot. Int. 11, 11–18. doi: 10.1093/heapro/11.1.11

[ref3] Applewhite A. (2016). This Chair Rocks: A Manifesto Against Ageism. Perfect Paperback. New York: Celadon Books.

[ref4] Araujo L. Ribeiro O. Paúl C. (2017). Hedonic and eudaimonic well-being in old age through positive psychology studies: a scoping review. Anal. Psicol. 33:568. doi: 10.6018/analesps.33.3.265621

[ref5] Ayalon L. Tesch-Römer C. (eds.) (2018). Contemporary Perspectives on Ageism: International Perspectives on Aging 19. Vol. 19. Cham, Switzerland: Springer Open.

[ref6] Bangen K. J. Meeks T. W. Jeste D. V. (2013). Defining and assessing wisdom: a review of the literature. Am. J. Geriatr. Psychiatry 21, 1254–1266. doi: 10.1016/j.jagp.2012.11.020, PMID: 23597933 PMC3896261

[ref39] Burns L. Masood M. (2018). “Storytelling: a medium for co-design of health and well-being services for seniors,” in Entertainment Computing–ICEC 2018. LNCS 11112. Lecture Notes in Computer Science. Vol. 11112. Springer International Publishing, 349–354.

[ref7] Carstensen L. L. (2011). A Long Bright Future: Happiness, Health, and Financial Security in an Age of Increased Longevity. New York: Public Affairs.

[ref41] Cary L. A. Alison L. C. Jessica R. (2017). The ambivalent ageism scale: developing and validating a scale to measure benevolent and hostile ageism. Gerontologist 57, e27–e36. doi: 10.1093/geront/gnw118 27520730

[ref8] Comincioli E. Chirico A. Masoodian M. (2021).“Improving the language of designing for ageing,” in Lecture Notes in Computer Science (Including Subseries Lecture Notes in Artificial Intelligence and Lecture Notes in Bioinformatics). Vol. 12933. Cham: Springer, 405–425.

[ref9] Coughlin J. F. (2017). The Longevity Economy: Unlocking the World’s Fastest-Growing, Most Misunderstood Market. New York: Public Affairs.

[ref10] Diener E. Seligman M. E. P. (2004). Beyond money: toward an economy of well-being. Psychol. Sci. Public Interest 5, 1–31. doi: 10.1111/j.0963-7214.2004.00501001.x, PMID: 26158992

[ref11] Directorate-General for Economic and Financial Affairs (2018). “The 2018 Ageing Report. Underlying Assumptions and Projection Methodologies.” Brussels.

[ref12] Ellis S. R. Morrison T. G. (2005). Stereotypes of ageing: messages promoted by age-specific paper birthday cards available in Canada. Int. J. Aging Hum. Dev. 61, 57–73. doi: 10.2190/ULUU-UN83-8W18-EP70, PMID: 16060333

[ref13] Fiske S. T. (2014). “Social beings,” in Core motives in social psychology. 3rd Edn. Wiley.

[ref14] Gaggioli A. Ferscha A. Riva G. Dunne S. Viaud-Delmon I. (eds.) (2016). “Transformative experience design,” in Human Computer Confluence Transforming Human Experience Through Symbiotic Technologies. De Gruyter, 97–122.

[ref15] Gendron T. L. Ayn Welleford E. Inker J. White J. T. (2016). The language of ageism: why we need to use words carefully. Gerontologist 56, 997–1006. doi: 10.1093/geront/gnv066, PMID: 26185154

[ref16] Greenwald A. G. Rudman L. A. Nosek B. A. Banaji M. R. Farnham S. D. Mellott D. S. (2002). A unified theory of implicit attitudes, stereotypes, self-esteem, and self-concept. Psychol. Rev. 109, 3–25. doi: 10.1037/0033-295X.109.1.3, PMID: 11863040

[ref17] ICAA (2011). ICAA’s Guidelines for Effective Communication with Older Adults. International Council on Active Aging. Available at: www.changingthewayweage.com

[ref18] Jacobson S. (2014). Personalised Assistive Products: Managing Stigma and Expressing the Self. Helsinki: Aaalto ARTS Books.

[ref19] Jönson H. (2013). We will be different! Ageism and the temporal construction of old age. Gerontologist 53, 198–204. doi: 10.1093/geront/gns066, PMID: 22555885

[ref20] Khaw D. Kern M. L. (2015). A cross-cultural comparison of the PERMA model of well-being. J. Psychol. 8, 10–23.

[ref21] Kliegel M. Iwarsson S. Wahrendorf M. Minicuci N. Aartsen M. J. (2020). The European journal of ageing at the beginning of the decade of healthy ageing. Eur. J. Ageing 17, 1–2. doi: 10.1007/s10433-020-00557-8, PMID: 32158367 PMC7040142

[ref22] Komp K. Aartsen M. (eds.) (2013). Old Age in Europe: A Textbook of Gerontology. Springer Briefs in Aging. New York, London: Dordrecht Heidelberg.

[ref23] Krekula C. (2009). Age coding—on age-based practices of distinction. Int. J. Ageing Later Life 4, 7–31. doi: 10.3384/ijal.1652-8670.09427

[ref24] Lee H. R. Riek L. D. (2018). Reframing assistive robots to promote successful aging. ACM Transact. Hum. Robot Interact. 7, 1–23. doi: 10.1145/3203303

[ref25] Levy B. Banaji M. R. (2002). “Implicit agesim,” in Ageism: Stereotyping and Prejudice Against Older Persons. ed. Nelson T. D. (Cambridge, Massachusetts: MIT Press).

[ref26] Levy B. R. Hausdorff J. M. Hencke R. Wei J. Y. (2000). Reducing cardiovascular stress with positive self-stereotypes of aging. J. Gerontol. B Psychol. Sci. Soc. Sci. 55, 205–213. doi: 10.1093/geronb/55.4.p205, PMID: 11584877

[ref27] Levy B. R. Slade M. D. Kunkel S. R. Kasl S. V. (2002). Longevity increased by positive self-perceptions of aging. J. Pers. Soc. Psychol. 83, 261–270. doi: 10.1037/0022-3514.83.2.261, PMID: 12150226

[ref28] Park G. H. M. Corn A. A. (2017). “Positive psychology,” in Applied Exercise Psychology. New York, NY: Routledge, 417–431.

[ref29] Seligman M. (2011). Flourish. A Visionary New Understanding of Happiness and Well-Being. New York, USA: Simon Schuster.

[ref30] Seligman M. E. P. (2019). Positive psychology: a personal history. Annu. Rev. Clin. Psychol. 15, 1–23. doi: 10.1146/annurev-clinpsy-050718-095653, PMID: 30525996

[ref31] Sholl J. Rattan S. I. S. (eds.) (2020). “Explaining health across the sciences,” in Healthy Ageing and Longevity. Vol. 12. Switzerland: Springer Nature, 368.

[ref32] Vauclair C. M. Hanke K. Huang L. L. Abrams D. (2017). Are Asian cultures really less ageist than western ones? It depends on the questions asked. Int. J. Psychol. 52, 136–144. doi: 10.1002/ijop.12292, PMID: 27374765 PMC5347948

[ref33] Vauclair C. M. Rodrigues R. B. Marques S. Esteves C. S. Cunha F. Gerardo F. (2018). Doddering but dear … even in the eyes of young children? Age stereotyping and prejudice in childhood and adolescence. Int. J. Psychol. 53, 63–70. doi: 10.1002/ijop.12430, PMID: 28474340

[ref40] Vines J. Gary P. Peter W. Patrick O. Katie B. (2015). An age-old problem: examining the discourses of ageing in HCl and strategies for future research. Tochi 22, 1–27. doi: 10.1145/2696867

[ref34] Voss P. Wolff J. K. Rothermund K. (2017). Relations between views on ageing and perceived age discrimination: a domain-specific perspective. Eur. J. Ageing 14, 5–15. doi: 10.1007/s10433-016-0381-4, PMID: 28804390 PMC5550618

[ref35] WHO (2006). Constitution of the World Health Organization. World Health Organization.

[ref36] WHO (2020). “Decade of Healthy Ageing 2020–2030.” Available at: https://www.who.int/docs/default-source/decade-of-healthy-ageing/final-decade-proposal/decade-proposal-final-apr2020-en.pdf?sfvrsn=b4b75ebc_5 (Accessed July 10, 2021).

[ref37] World Health Organization (2021) “Ageing and Health.” World Health Organisation. Available at: https://www.who.int/news-room/fact-sheets/detail/ageing-and-health (Accessed January 2, 2021).

[ref38] Zlatev J. Blomberg J. (2015). Language may indeed influence thought. Front. Psychol. 6:1631. doi: 10.3389/fpsyg.2015.01631, PMID: 26582997 PMC4628110

